# Manual Therapy versus Localisation (Tactile, Sensory Training) in Patients with Non-Specific Neck Pain: A Randomised Clinical Pilot Trial

**DOI:** 10.3390/healthcare11101385

**Published:** 2023-05-11

**Authors:** Eleftheria Thomaidou, Christopher James McCarthy, Elias Tsepis, Konstantinos Fousekis, Evdokia Billis

**Affiliations:** 1 Department of Physiotherapy, School of Health Rehabilitation Sciences, University of Patras, 26504 Patras, Greecebillis@upatras.gr (E.B.); 2 Manchester Movement Unit, Manchester School of Physiotherapy, Manchester Metropolitan University, Manchester M15 6BH, UK; chris.mccarthy@mmu.ac.uk

**Keywords:** neck pain, localisation, tactile sensory training, manual therapy

## Abstract

Manual therapy (MT) techniques typically incorporate localised touch on the skin with the application of specific kinetic forces. The contribution of localised touch to the effectiveness of MT techniques has not been evaluated. This study investigated the immediate effects of MT versus localisation training (LT) on pain intensity and range of movement (ROM) for neck pain. In this single-blind randomised controlled trial thirty eligible neck pain volunteers (23 females and 7 males), aged 28.63 ± 12.49 years, were randomly allocated to MT or to a motionless (LT) group. A single three-minute treatment session was delivered to each group’s cervico-thoracic area. The LT involved tactile sensory stimulation applied randomly to one out of a nine-block grid. Subjects were asked to identify the number of the square being touched, reflecting a different location on the region of skin. MT involved three-minute anteroposterior (AP) glides and sustained natural apophyseal glides (SNAG) techniques. Pre- and post-intervention pain intensity were assessed using a pressure pain threshold (PPT) algometer and the numeric pain rating scale (NPRS). Neck ROM was recorded with a bubble inclinometer. Improvements in ROM and self-reported pain were recorded in both groups (*p* < 0.001) without differences in NPRS, ROM or PPT scores between groups (*p* > 0.05). Tactile sensory training (localisation) was as effective as MT in reducing neck pain, suggesting a component of MT’s analgesic effect to be related with the element of localised touch rather than the forces induced during passive movements.

## 1. Introduction

Neck pain (NP) is one of the major public health musculoskeletal problems in modern society, which has a great impact on people’s lives, with high prevalence and occurrence rates [1,2] leading to global disability [2]. At the same time, the economic burden of neck pain is remarkable and its associated treatment costs are continuously increasing [3]. In 2016, among the 154 conditions, neck pain had the highest health care spending in the United States [3]. Interestingly, substantial evidence indicates that neck pain represents a significant contributing factor to medical care seeking, disability, reduced work productivity and work absenteeism due to sickness [4].

Evidence suggests that patients suffering from chronic pain present structural and functional alterations in brain imaging such as in fMRIs [5]. The area of the brain which is mainly affected in relation to pain experience is the primary somatosensory area (S1). Within the brain there is a virtual representation of the body, which is labeled as the cortical homunculus. The homunculus is located on the somatosensory cortex on the postcentral gyrus of the anterior parietal lobe and is associated with sensation. Flor in 2003 [6] has found that the cortical representation of the homunculus can be modified, depending on the sensory input. Likewise, changes in the cortical homunculus can be displayed in the event of increased input due to training as well as in cases of loss of input due to differentiation [6]. The results pooled by a systematic review and metanalysis showed that participants suffering from chronic pain demonstrated altered tactile acuity, whilst the two-point discrimination (TPD) threshold was increased. The lack of ability to determine the sense of touch (loss of tactile acuity and increased TPD) is dependent not only on reduced tactile detection [7], or impairment in transmission via neural pathways [8], but also on a manifestation of altered somatosensory processing. Thus, persistent pain was associated with cortical disruptions of the painful area [9]. Noticeably, a recent study of Harvie et al. (2018) has shown that tactile acuity is affected in people suffering from chronic neck pain [10]. More specifically, the impairment in discriminative ability is thought to reflect cortical changes in the neurons and their synaptic mechanisms in the primary somatosensory cortex (S1) of the painful body part [6].

A review of the literature has shown that the delivery of manual therapy, which is classically considered to be a “bottom-up” or a “hands-on” approach, is beneficial for patients suffering from neck pain [11]. It is demonstrated that manual therapy can be beneficial for immediate and short-term improvements in range of motion, pain levels and function, both in acute and chronic pain patients as well as those with whiplash [12]. Inherent in the application of MT is an element of touching the skin, thereby inducing local, tactile sensory input to the central nervous system. In addition to localised touch, MT typically involves the introduction of kinetic forces inducing movement [13]. Localisation training (LT), as a new “top down” approach, which involves repeated, localised, tactile sensory stimulus without physical movement of the skeletal system, is thought to “sharpen” or “refocus” the sensory homunculus’ representation of the body part and has been associated with improvements in movement and decreases in pain [7,14]. The area of cortex spatially reflecting a map of the physical body is the primary somatosensory cortex (S1) [15,16]. It has been reported that participants suffering from pain demonstrate disorganised representation of the painful region in S1, compared to healthy individuals [7,17].

Το date, only limited pathological conditions have been investigated in terms of tactile acuity and cortical reorganization, such as low back pain [9], phantom limb pain [18], complex regional pain syndrome [7] and neck pain [10], and whilst the positive effects of sensory training have already been suggested [7,18,19], there is little evidence on the upper spinal area. Despite this growing interest, to the best of our knowledge, there is a lack of consensus about the effectiveness of tactile sensory treatment (localisation) and the difference of interventional effect between manual therapy and localisation in patients suffering from neck pain. In order to begin to unpick the therapeutic elements of MT this study aimed to explore the effects of localised touch compared to traditional MT techniques (which could be considered to be localised touch plus kinetic force application) on pain intensity and mobility of the neck. 

## 2. Materials and Methods

A single-blind randomised clinical pilot trial was conducted at the Physiotherapy Department of the University of Patras. The trial was registered on the international ISRCTN registry with study ID ISRCTN37282752. 

### 2.1. Subjects

Recruitment was conducted via social media, local newspaper adverts or via direct medical referral to physiotherapy. Eligible subjects who already had a medical diagnosis for non-specific neck pain were invited to participate in the study provided that they had neck pain at the time of the study and for at least 1 week prior to their recruitment. Participants were excluded if they could not read or understand spoken/written Greek, were under the age 18 or over 65, had undergone spinal surgery in the area, had any skin condition preventing them from receiving tactile stimuli, had any contraindications to manual therapy, such as vertebral artery insufficiency, spinal instability, steroid medication use, malignancy [20] or presented with any symptoms related to neurological conditions altering sensation (i.e., peripheral neuropathy, multiple sclerosis, diabetes, diagnosis of radiculopathy, or cervical myelopathy). All participants were required to complete a health questionnaire, ensuring that each participant was clear of contraindications, “red flags” and any other additional factors included in the exclusion criteria [21]. Given the absence of red flags, no imaging was required or indicated according to relative guidelines [22]. 

### 2.2. Sample Size

As there was no previous research available to suggest the minimum number of subjects required for adequate statistical power to detect a treatment effect, this study was considered to be a pilot, which would enable power calculations to be undertaken post hoc. Based on previous studies in different areas of focus which have applied tactile sensory training, between 15 and 60 participants have been used [10,23,24,25,26]. Thus, a convenience of 30 participants was used. Prior to participation in the study, informed consent took place where all subjects were able to ask questions before their written consent, according to the ethical standards of the Declaration of Helsinki. The researcher explained the procedure and participants were reminded at this point that they were free to withdraw from the study at any time. 

### 2.3. Randomization

Participants were randomly assigned into either the LT group or MT group using an online randomisation program (www.randomizer.org). 

### 2.4. Blinding

The participants’ blindness to group allocation was ensured as participants were not aware of the exact hypotheses of the research. Participants were made aware that the research was a comparison of two treatment techniques. Treatment and assessment were undertaken by different physiotherapists.

### 2.5. Trial Registry

The trial was registered with the ISRCTN registry with the study ID ISRCTN37282752. Ethical approval was provided by the University of Patras ethical committee.

### 2.6. Outcome Measures and Measurements

Self-reported neck and thoracic pain intensity was evaluated using the 0–10 numeric pain rating scale (NPRS), as it is commonly used in various spinal pain studies [27] and in clinical practice. Cleland et al., in 2008 [28], found that the minimum detectable change (MDC) and minimal clinically important difference (MCID) for the NPRS to report a true difference were 2.1 and 1.3 points, respectively, in participants with mechanical neck pain. 

**Figure 1:**
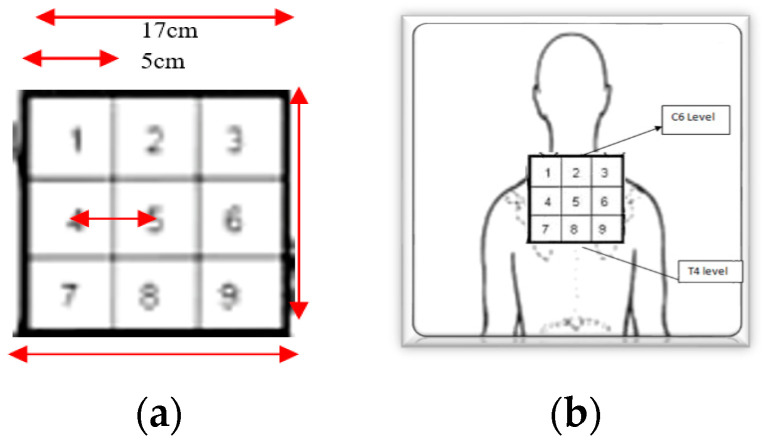
(**a**) Localisation 9-block grid design; (**b**) application of the 9-block grid in the cervicothoracic area.

Pain pressure threshold (PPT) is defined as the minimal amount of pressure for which a stimulus is perceived as painful, measured through an algometer kg/cm^2^, and it is also used to objectively measure pain. Potter et al. in 2006 [29] found algometry a stable and ideal tool to objectively measure an individual’s pain. Chesterton et al. [30] concluded that pressure algometry, when used with the correct technique, is a highly reliable measurement of PPT. The minimum detectable change (MDC) for PPT to report a true difference in the upper trapezius muscle in subjects with NP has been determined as 47.2 kPa = 0.48 kg/cm^2^ [31]. An analogue handheld algometer (Wagner FDX-20 device) with a surface area of 1 cm^2^ at the round rubber tip was applied vertically to the skin at a rate of 1 kg/cm^2^/s. After providing standardized instructions, the examiner applied force via the algometer until the participant felt that pressure sensation turned into an unpleasant feeling, the participant stated “stop” and the score was noted. With the patient seated, and a 3 × 3 grid positioned on the skin, three points were marked on the participants’ skin with a pen to ensure that the second measurement would be recorded from the same area as the first to increase reliability. The PPT of the participants was assessed by the same examiner. Two measurements (pre- and within 1 min immediately post-intervention) were recorded at the same 3 points marked on the participant’s skin and in the same order: starting centrally (5th square of the grid), left (4th square) and ending right (6th square) (Figure 1).

The cervical range of motion (ROM) was measured (pre- and within 1 min post-intervention) using a baseline bubble inclinometer. Bubble inclinometry has been shown to be a reliable method of measuring cervical range of movement, with ICC values ranging from 0.80 to 0.93 [32]. Prior to neck ROM measurements, all patients were requested to carry out all neck movements to familiarize themselves with the procedure. Patients were given consistent verbal instructions and were asked to perform active movement as pain-tolerated or as to the full extent of mobility in flexion, extension, left and right lateral flexion (in a sitting upright position with the arms relaxed on the plinth, feet on the floor horizontally and head in a neutral position). For measuring flexion and extension ROM, the assessor positioned the bubble goniometer in the sagittal plane in the midline of the participant’s head. The patient had a starting point in the middle position (corrected by the therapist if needed). Correspondingly, for the measurement of lateral flexion the bubble goniometer was positioned in the frontal plane. Left and right cervical rotations were performed with the patient lying supine and the bubble inclinometer positioned between the eyebrows in the center of the participant’s forehead. These movements were performed three times each; the scores were recorded and averaged.

The Neck Disability Index (NDI) is a patient-completed, condition-specific functional status questionnaire with 10 items, including pain, personal care, lifting, reading, headaches, concentration, work, driving, sleeping and recreation, to evaluate disability in patients with neck pain [33]. Each section is scored from 0 to 5 and the total score is expressed as a percentage [33]. The NDI has sufficient support and usefulness to retain its current status as the most commonly used self-reported measure for neck pain internationally. It is a valid and reliable tool, designed to measure function, and it has been translated into Greek with high internal consistency (Cronbach alpha: 0.85) and excellent test-retest reliability (ICC: 0.93) [34]. The NDI was used only at baseline to estimate the amount of disability of each subject. Due to the short-term treatment utilized in the study, the NDI could not be justified in having changed the sample’s disability levels during a treatment session.

The Hospital Anxiety and Depression Scale (HADS) has been administered to provide clinicians with an acceptable, reliable, valid, practical tool for identifying and quantifying depression and anxiety and has also showed evidence of reliability and validity in population with neck pain [35]. The HADS questionnaire has been translated and widely used in over 25 countries and consists of 14 questions rated on a 4-point Likert scale (range 0–3). It is designed to measure anxiety and depression (7 items per subclass) and the score is obtained for each subcategory separately but also from the sum of all 14 questions. In this clinical study the validated Greek version by Michopoulos et al. [36] was used, which has shown good psychometric properties.

### 2.7. Assessors

The main researcher of this study (ET), a registered physiotherapist with fifteen years of clinical experience, explained the procedure to the participants and collected the information at baseline. All the measurements and data recording pre- and post-intervention were performed by the main researcher. The therapist who applied the localisation intervention to the tactile sensory training group and the manual therapy intervention was the project supervisor (EB), who is a manual therapist with more than 20 years of clinical experience.

### 2.8. Interventions

#### 2.8.1. Manual Therapy (MT) Group

Participants received a single session of MT, lasting a total of three minutes of contact time, including a one-minute anteroposterior (AP) gliding technique at the C7-T1 vertebral segment and two minutes sustained natural apophyseal glides (SNAGs) into rotation at T1–T2 and T3–T4 levels. With the subject lying supine, the therapist applied AP mobilization, as described by McCarthy [13] to the anterior surface (articular pillar) of the low cervical vertebrae on the painful side, Grade III for one minute, at a frequency of 2 HZ (using a metronome). Then, with the patient in a sitting position, the therapist located the T1–T2 level and placed her hand unilaterally. The participant was asked to actively rotate to the right for 30 s and to the left for 30 s (10 times each side) while the therapist guided the glide through the movement, thus performing SNAGs [37,38]. The procedure was then repeated lower down the thoracic spine (T3–T4). The force was applied parallel to the facet plane [37]. The SNAG technique was chosen, as it is suggested for painful movement dysfunctions and, in contrast to other manual therapy techniques, is performed with the spine under normal load-bearing conditions. Further, it includes active and passive elements of physiological movements with accessory glides, within the available ROM, and it is under the patient’s control. The duration of the treatment was based on previous studies investigating the effects of MT on pain and ROM in patients suffering from neck pain [39,40,41].

#### 2.8.2. Localisation Training (LT) Group

A nine-square, 3 × 3 grid was designed for the experimental procedure using cardboard. The size of the individual squares within the grid was based on the minimal two-point discrimination in the neck area. This is reported to be 4.59 cm [42]. Therefore, the distance between the middle of the individual squares was greater than the minimal two-point discrimination figures, at 5 cm (Figure 1). The localisation training was performed for 3 min, in order to provide the equivocal contact time as the MT group treatment duration.

**Figure 2:**
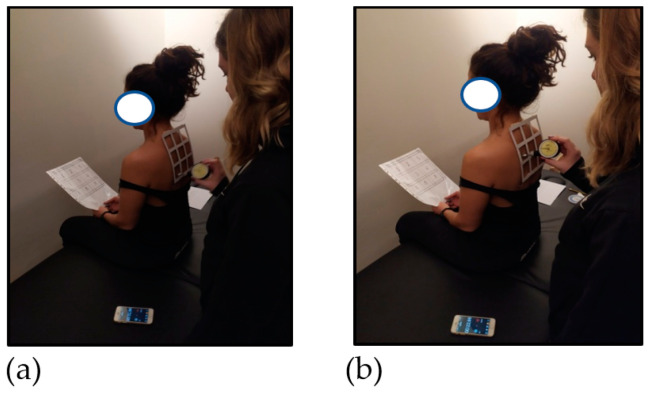
(**a**,**b**) Tactile sensory (localisation) training.

Participants were seated with the grid held against the skin between C6 and T4 (Figure 1b). The participants were taught where each square was by having it touched and its number being announced by the therapist. Subsequently, whilst the therapist randomly touched squares, the participant was requested to answer which number was being touched. With a successful identification of the individual square, the therapist proceeded to another square on the grid. In the event of an incorrect response, the same grid was touched again and its number announced, helping the patient to improve their acuity in location (Figure 2a,b).

### 2.9. Data Analysis 

In order to evaluate the intra-rater, test-retest reliability of the measures of ROM and PPT, a pre-pilot was undertaken (*n* = 20), where measurement error was evaluated using Intraclass Correlation Coefficients (ICC_2,1_) via a two-way random model with 95% CIs around the point estimates.

A Shapiro–Wilk test was conducted to determine if data was normally distributed. The non-parametric normative data was tested for statistical significance utilizing a Wilcoxon statistical test, for within group, and/or a Mann–Whitney U test, for between groups. When the assumptions for parametric testing were met, an independent samples *t*-test was performed between the two groups and the paired sample *t*-test was conducted for the within-group comparisons of the measurements before and after the interventions. A two-way mixed analysis of variance (ANOVA) was performed for the comparison of interaction of time (pre- and post-treatment) and group (localisation and manual therapy group). Statistical significance was accepted at *p* < 0.05. Statistical testing was carried out using the IBM Statistical Package for Social Science (SPSS) Software (version 24, Chicago, IL, USA). 

## 3. Results

### 3.1. Participants

**Figure 3:**
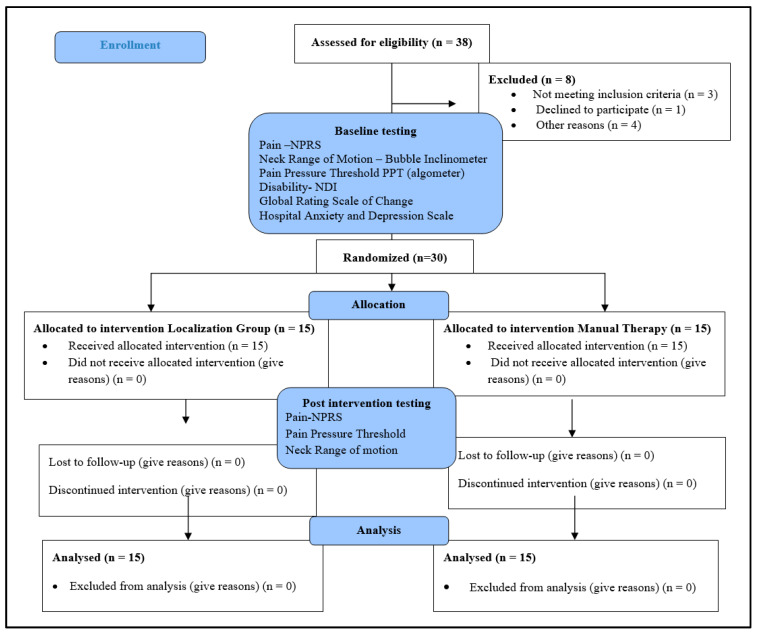
A CONSORT Flow diagram showing participant flow in the study.

A sample of 30 participants (23 females and 7 males) aged 28.63 ± 12.49 years (ranging between 18 and 64 years) with non-specific neck pain who met the inclusion criteria were randomly assigned into two equal groups (*n* = 15); the LT group (11 females, 4 males aged 26 ± 10 years) and the MT group (12 females, 3 males aged 31 ± 15 years). Figure 3 illustrates the CONSORT flow chart diagram. There were no differences in participants’ demographics (age, height, weight or BMI), in clinical characteristics such as duration of pain, or in measurements/scores of the NPRS, ROM, PPT, NDI or HADs between the groups at baseline (*p* > 0.05) (Table 1, Table 2 and Table 3).

### 3.2. Within Group Analyses

Statistical analyses pre- and post-intervention were conducted using the localisation group data to investigate the effectiveness of time in the dependent variables of the NPRS, PPT and neck ROM. There is evidence to suggest that “my pain now” was less post-treatment, 3.73 (±1.58), than pre-treatment, 4.93 (±1.33), in the localisation group (*p*-value = 0.012). With regard to PPT, the paired *t*-test demonstrated a numerical decrease in the pain pressure threshold in the localisation group, but the changes were not statistically significant (*p* > 0.05). The results show that neck ROM improved in all directions. Most of the movements had an increase of more than 3° between pre- and post-intervention evaluations, which was still not enough for a detectable change in bubble inclinometer.

Statistical analyses pre- and post-intervention were conducted using the manual therapy group data to investigate the effectiveness of time in the dependent variables of the NPRS, PPT and neck ROM. There is evidence to suggest that “my pain now” was less post-treatment, 3.20 (±1.26), than pre-treatment, 4.33 (±1.45), in the manual therapy group (*p*-value = 0.003). With regard to PPT, the analysis did not reveal any statistically significant changes (*p* > 0.05). Neck range of motion also improved in all directions (*p* < 0.001). Most of the movements had an increase of more than 4° between pre- and post-intervention evaluations, which is not enough for a clinically meaningful change.

### 3.3. Between Group Analyses

Following one session of treatment, all participants were reassessed for their pain levels, as measured by the NPRS, to define their present pain which was improved regardless of the type of treatment. The average “pain intensity now” after participants had received manual therapy was less, 3.20 (±1.26), than when they received tactile sensory (localisation) training, 3.73 (±1.58). However, an independent *t*-test showed that the significance level was *p* = 0.389, which is above the accepted level of significance (α > 0.05); thus, the difference is not statistically significant between the localisation and manual therapy groups. In both intervention groups the self-reported level of pain was decreased, and the mean change was statistically significant (*F* statistic (*p*-value) 22.80 (0.000 **). Irrespective of the intervention groups, the reported reductions in neck pain identified by the NPRS were less than 2.1 and thus deemed to provide clinical significance (Table 3).

The independent *t*-test analysis confirmed that there is no significant statistical difference between the PPT values before treatment across the two groups (*p* > 0.05). Similarly, PPT measured at 3 points (centrally, right and left) after the intervention between groups, showed no significant difference, with significance level at 0.605, 0.367 and 0.629, respectively, (all *p* > 0.05) (Table 3). 

Neck ROM in all directions was improved from baseline to post-treatment in both groups (*F* = 12.54, *p* < 0.002). However, between groups, the improvement shown was not statistically significant post-intervention for rotation, lateral flexion and extension (*p* > 0.05) but was in favor of the manual therapy group for flexion (*p* = 0.030) with a very small, clinically unimportant increase (Table 3).

### 3.4. Within-Subjects Interaction Time * Group Analysis

A two-way mixed ANOVA was conducted to investigate the interaction between time and group and was found to be statistically significant only for lateral flexion left (*F* = 5.72, *p* = 0.024 *) and extension (*F* = 4.24, *p* < 0.05 *), favoring the manual therapy group. The two-way mixed ANOVA did not reveal any statistically significant differences for present pain intensity (*p* > 0.05), PPT (*p* > 0.05) or other neck ROM directions (*p* > 0.05). Table 4 summarizes the significance values of the primary outcome measures, the NPRS, range of motion and PPT, assessed by a two-way mixed ANOVA test, and graphs illustrate the changes in the above-mentioned outcome measures in both groups. More specifically, the improvement in lateral flexion left (from M = 37.220 SD = 9.39 pre-intervention to M = 42.40, SD = 9.11 post-intervention) in the manual therapy group was more statistically significant than in the localisation group (from M = 38.70, SD = 6.96 pre-intervention to M = 40.90, SD = 6.60). In extension the improvement in the manual therapy group (from M = 46.7560, SD = 10.78 pre-intervention to M = 50.960, SD = 9.04 post-intervention) was statistically greater than in the localisation group (from M = 52,220 SD = 9.62 pre-intervention to M = 53.330, SD = 9.17 post-intervention).

## 4. Discussion

This is the first randomised clinical trial investigating the effects of manual therapy versus localisation training on pain and neck mobility in participants with non-specific neck pain. In the present study, the results suggest that a single session of tactile sensory (localisation) training in the cervicothoracic area resulted in similar outcomes to a single session of manual therapy. Both groups demonstrated small and equivocal improvements in levels of pain and neck mobility immediately post-intervention. This data is in line with the findings of studies investigating MT [39,43,44] and localisation training in low back pain [45], reflecting the fast-acting anti-nociceptive effects of many forms of touch. 

Improvements in pain and range of movement were observed in both groups but were small, and this study was unable to identify any clinically significant differences in pain perception and neck mobility between the two interventions. 

Existing knowledge suggests that the mechanisms of action of manual therapy include a mix of three mechanisms—biomechanical, neurophysiological and/or placebo effects [46,47]. The mechanisms by which manual therapy improved ROM can be attributed mainly to mechanical effects, such as a change in the length of connective tissue structures-ligaments, facets’ joints capsule and muscles-stretching adhesions. The reason for this could be the short duration of treatment on moderate patients’ levels of pain. The observed improvement in cervical ROM followed the pattern described in the literature after manual therapy using mobilizations in patients with NP. Likewise, the magnitude of changes in ROM of the above-mentioned studies varies, and it could be influenced by the type and method of the applied manual therapy technique. 

The reduction of pain levels shown in the manual therapy group is probably due to biomechanical effects while normalizing the muscle activity and stretching the joint tissues, neurophysiological effects while stimulating mechanoreceptors and psychological effects of mobilization [43,46]. One possible explanation to understand the impact of the application of mobilization techniques to the cervicothoracic area on pain relief in NP subjects has been the principle of regional interdependence. According to this principle, the subject’s pain may be related to a restriction in a proximal or a distal anatomical area [48], which might also support the present observations. The decrease in pain intensity in the manual therapy group in our study was statistically (*p* = 0.003) but not clinically significant (<MDC).

No clinically significant hypoalgesic effects were shown in the current study. These findings did not agree with previous studies where the results of a double blinded RCT indicated that the anteroposterior cervical mobilization technique grade III produced a hypoalgesic effect, as revealed by increased pressure pain thresholds on the side of treatment [43]. A potential explanation could be that the sample size was underpowered to detect changes in PPT over all points because estimation was only based on available data for the cervical spine. This could also explain why the current study observed changes in neck pain intensity but not in PPT. It should be noted that in the present study, patients only received one session of the intervention. Perhaps more MT sessions are necessary to experience a cumulative effect when dealing with manual therapy techniques directed at the thoracic spine.

The exact physiological mechanism for the analgesic effect of tactile sensory training (localisation) is unclear. However, theories suggest that as the training includes not only a component of localised, sensory stimulation but also an active process of focused, perception and requirement for accurate identification of body location, there is a concomitant process of cortical reorganization in the primary somatosensory cortex [17]. Although the exact mechanisms relating S1 remapping and pain relief remains a matter of conjecture, a possible explanation might be that stimulus over the skin associated with attention and localisation tend to activate mechanical receptors (i.e., Meisner corpuscles and Merkel’s discs), thus providing a sensory input to the CNS, stimulating the fast myelinated A beta fibers and inhibiting the transmission of nociceptive signals through slow, nonmyelinated C fibers, resulting in an analgesic effects [49]. These changes are observed with the introduction of a cognitively demanding task and result in a process of physiological learning, cortical “tuning” to the body region, spatial awareness, proprioception and pain modulation [14,50]. The proposed mechanism of action underlying this phenomenon could be the cortical reorganization through cognitive educational approach. The tactile sensory training involves the patient concentrated, attempting to identify the location and type of stimulus applied by the therapist and receiving feedback on correctness; thus, educating the patient. In other words, tactile sensory training is a mentally challenging approach, for which concentration and continuous feedback raises attentional control and positively influences pain and movement [51]. Finally, authors have suggested that passive techniques, such as the localisation technique may have a placebo effect and symptom improvement. The explanation for the short-term analgesia effect of placebo is associated with opioid substance release [52], occurring through tactile sensory stimulation.

If the focusing of attention to localised touch has the potential to reduce spinal pain, what does the addition of passive movement contribute to the analgesic effect? One might have expected that a localised application of touch, combined with the additional component of passively generated movement, would provide a greater input to the nervous system and thereby induce more significant descending inhibitory effects on pain. This was not the finding of our data. 

Several manual therapy studies that have shown equivocal reductions in spinal pain perception in groups receiving localised touch, combined with a localised kinetic force applied to a specific, intervertebral level when compared to a group receiving general, nonlocalised movement of a region of the spine. However, none of the studies included an element of localisation training of sensory attention to the location of the touch [53,54,55]. This has led some authors to question the need to target localised points, within painful regions of the spine, for the application of forces inducing passive movement [55,56]. Thus, the application of localised touch and local movement to the spine of a recipient, not focusing their attention on a localised tactile stimulation, is not superior to non-specific regional touch and regional movement. This suggests that whilst both applied passive movements and localisation training of sensory attention can reduce spinal pain, the incorporation of an active learning process of identification of the painful body part’s position in space, facilitated by tactile localisation training, may enhance analgesic effectiveness.

However, the findings of the present study should be evaluated in light of its limitations. Our patient baseline data revealed low initial self-reported pain and disability scores that may have limited the available improvement via a floor effect. A single treatment session, used in this study, as well as the duration of the treatment (3 min) was not representative of typical treatment programmes for neck pain but was a preliminary assessment of immediate influence. Thus, we do not know the long-term influence on the between-group comparisons. The study was underpowered and based on the current study’s data a sample size of approximately 280 would be necessary to ensure adequate power to reduce erroneous acceptance of the null (*n* = 280, β = 0.2, α = 0.05). 

It is recommended that future research in this area should employ randomised controlled trials ensuring double-blinding and adequately sized sampling. It will also be important to collect long-term follow-up data to facilitate the development of treatment protocols. 

## 5. Conclusions

This is the first randomised clinical pilot trial investigating the immediate effects of manual therapy versus tactile sensory training (localisation) on pain and neck mobility in participants with non-specific neck pain. The results of this study suggest that a single session of tactile sensory training (localisation) can be as effective as manual therapy in reducing neck pain and improving neck ROM among participants with neck pain. Tactile sensory training (localisation) can be included in multi-modal rehabilitation programmes for participants with neck pain. The combination of the application of passive kinetic forces that induce spinal movement (as applied with manual therapy) in addition to the facilitation of an active cognitive process of focused attention (to the localised tactile stimulus, facilitating proprioception and spatial awareness) may prove to be more efficacious than each component in isolation. If this is the case, there would be scope for the development of treatment approaches incorporating tactile stimulus/passive movement with an active, focused, attention on body position and location in space. 

## Figures and Tables

**Table 1:** Baseline demographics between the Localisation and the Manual Therapy groups.

	Whole Sample (*n* = 30)	Localisation Group (*n* = 15)	Manual Therapy Group (*n* = 15)	Significance (*p*-Value)	
	Mean ± SD				
Age (years)	28.63 ± 12.49	26.4 ± 9.66		30.87 ± 14.81	0.461
Height (m)	1.74 ± 0.099	1.71 ± 0.09		1.70 ± 0.11	0.746
Weight (kg)	78.5 ± 15.40	72.26 ± 17.80		70.6 ± 13.17	0.713
BMI (kg/m^2^)	24.61 ± 3.61	24.40 ± 3.99		24.33 ± 3.33	0.958

SD = standard deviation.

**Table 2:** Disability, anxiety and depression levels across Localisation and Manual Therapy groups.

	Whole Sample (*n* = 30)	Localisation Group (*n* = 15)	Manual Therapy Group (*n* = 15)	Significance (*p*-Value)
	Mean ± SD			
NDI score (%)	21 ± 0.9	22 ± 1	19 ± 0.8	0.359
HADs (Total)	11.83 ± 5.91	12.07 ± 6.11	11.60 ± 5.91	0.838
HADS-Anxiety	7.97 ± 3.92	8.07 ± 4.15	7.87 ± 3.81	0.902
HADS-Depression	3.87 ± 2.67	4 ± 2.62	3.73 ± 2.81	0.624

NDI = Neck Disability Index, HADS = Hospital Anxiety and Depression Scale.

**Table 3:** Baseline and post-intervention outcomes between Localisation and Manual Therapy Groups.

	BASELINE				POST-INTERVENTION		
	Localisation Group	Manual Therapy Group	*t*-Test		Localisation Group	Manual Therapy Group	*t*-Test
	Mean (±SD)		*p* Value		Mean (±SD)		*p* Value
Pain intensity (NPRS)	4.93 (±1.33)	4.33 (±1.45)	0.345		3.73 (±1.58)	3.20 (±1.26)	0.389
PPT levels							
Central (5th square)	4.42 (±1.28)	3.77 (±1.17)	0.161		4.06 (±1.47)	3.80(±1.24)	0.605
Right (6th square)	4.09 (±1.51)	3.79 (±1.18)	0.542		4.07 (±1.47)	3.71 (±1.24)	0.367
Left (4th square)	4.17 (±1.59)	3.68 (±1.08)	0.337		4.10 (±1.45)	3.87 (±1.06)	0.629
ROM (°)							
Right Rotation	75 (±5.70)	75 (±7.25)	0.824		78 (±4.44)	79 (±5.42)	0.384
Left Rotation	71 (±10.45	73 (±7.58)	0.478		75 (±9.25)	77 (±7.97)	0.616
Right Lateral Flexion	42 (±5.55)	40 (±8.59)	0.270		47 (±5.88)	45 (±8.95)	0.379
Left Lateral Flexion	39 (±6.97)	37 (±9.39)	0.620		41 (±6.60)	42 (±9.11)	0.612
Flexion	45 (±9.29)	51 (±8.59)	0.108		47 (±8.06)	54 (±8.53)	0.030 *
Extension	52 (±9.62)	47(±10.79)		0.154	53 (±9.17)	51 (±9.04)	0.480

NPRS = numeric pain rating scale, PPT = pressure pain threshold, and ROM = range of movement. * Statistically significant value (*p* < 0.05).

**Table 4:** Two-way mixed ANOVA results, factor = time between localisation and manual therapy groups.

	Interaction Time * Group *F* (*p* Values)
NPRS now	0.02 (*p* = 0.892)
PPT central	3.21 (*p* = 0.084)
PPT right	0.07 (*p* = 0.793)
PPT left	1.89 (*p* = 0.180)
ROM Rotation R	0.48 (*p* = 0.495)
ROM Rotation L	0.29 (*p* = 0.595)
ROM Lateral Flexion R	0.07 (*p* = 0.794)
ROM Lateral Flexion L	5.721 (*p* = 0.024 *)
ROM Flexion	1.34 (*p* = 0.256)
ROM Extension	4.24 (*p* = 0.049 *)

* Statistically significant value (*p* < 0.05).

## Data Availability

The data that support the findings of this study are available from the Clinical Rehabilitation and Research Laboratory, Physical Therapy Department, University of Patras, and are not publicly available.
